# What Every Trained Nurse Should Know

**Published:** 1918-01-12

**Authors:** 


### NURSING AFFAIRS IN IRELAND.

### What Every Trained Nurse Should Know.

### Enemy War-Cries.

WHEN Germany appealed to the neutral world to compel England to grant to other nations "the freedom of the seas," British citizens rubbed their eyes with wonderment, and inquired what it all meant. To this hour no man has supplied an answer unless, perhaps, that Germany wants freedom for piracy. It is an enemy war-cry, false in its essence, and intended to appeal to ignorant prejudice. In the world of nursing, petty by comparison, these crafty German tactics have been emulated without scruple, and war-cries as meaningless as that of "the freedom of the seas" are trumpeted forth without explanation or argument, with the object of stirring up faction amongst the rank and file of the profession. I have already endeavoured to demonstrate that British nurses can never in any circumstances achieve or accomplish anything unless they are a solid body with a clearly defined programme comprising the essentials of progress. This simple and obvious truth is by no means adequately realised. If and when it is, the promoters of faction will be scouted as traitors. The meeting recently held in Dublin to which I recently referred was in itself an unimportant incident, scarcely noticed by the Irish press. But it was a perfect reflex of a general policy persistently pursued elsewhere, and therefore I propose to attempt what the Hon. Albinia Brodrick neglected—an analysis of the actual facts in which her mischievous and misleading war-cries find their origin.

### The V.A.D.s.

Miss Brodrick referred to the College of Nursing as "the College of V.A.D.s." She smiled: others smiled—and without further apology or explanation she lightly stepped along. Such strategy exactly corresponds with the German demand for "the freedom of the seas," and is intended, not to convince, but to create an unfavourable impression. One might imagine from the tone in which the V.A.D. problem is referred to by Miss Brodrick and her school that the College of Nursing is responsible for having called these excellent people into being. A very different tale, however, remains to be told. A fundamental and unalterable fact, which should be borne in mind by every nurse, trained or in training, is that **whether State Registration ever becomes an Act of Parliament or not, the V.A.D. is and will remain perfectly free, and competent according to law, to practise nursing for gain.** This is an irrefutable fact of far-reaching significance. Miss Brodrick and her friends may rail at the V.A.D.s, but the question faces them none the less—are the floodgates to be opened without discrimination when the war ends, and is the regular nursing profession to be thereby swamped? It is not any part of my duty here and now to discuss the national and Imperial value of the work of the V.A.D.s. Suffice it to say that it forms a valuable feature of British civilian effort to win the war, and when the conflict ends these women will carry with them into private life a large measure of the nation's gratitude. Some of them will have performed three years' duty, others three weeks'. Some will have been engaged on important work: others merely washing and scrubbing. It is most desirable in these circumstances that every trained nurse should realise that the three weeks' worker may, if it so please her, describe herself as a nurse, wear any uniform that she may fancy, and in every respect enter into full competition with women holding high hospital credentials. No law now existing, or to be enacted in the future, will prevent her from adopting such a course.

### The Attitude of the College.

So far, the College of Nursing has made no official pronouncement concerning V.A.D.s. If any should doubt the truth of this, the Regulations and Prospectus are open for inspection, and from these it is manifest that its policy and propaganda are directed altogether and exclusively to the unification and betterment of trained nurses. But in the Articles of Association, which define the limits of its powers, the College reserves the right, if its Board so decides, to classify the V.A.D.s and to make regulations for their further training and examination, and by this means to protect the regular profession, rather than leave it in such chaos as must of necessity ensue if a *laissez-faire* policy is pursued. If State Registration were to have the effect of making it illegal for an unregistered nurse to practise for gain, no action of this kind would be necessary. The passing of such an Act would automatically put the untrained out of business. But, as has already been shown, this is a wholly impossible proposition, and, in the circumstances, the practical nurse will naturally ask herself which is the better policy—to face the V.A.D. problem boldly, or to contemplate it as an ostrich contemplates its pursuers, with head buried in the sand?

As I have already pointed out, the College has, so far, made no pronouncement; whether it will make any training concessions to V.A.D.s or not is in the lap of the gods. But in my opinion it will be an act of folly to ignore their existence as though by so doing they could be obliterated. Hundreds of them are women of high education and intelligence. They have been engaged on hard hospital duty—they have become familiar with sickness and wounds, and to some extent trained in hospital routine and subjected to strict discipline. When the war is over, if they desire to continue their careers as nurses any hospital may admit them as probationers, and give them the training and facilities necessary to enable them to pass the examinations and become fully trained nurses in the ordinary course.

Now, instead of indulging in flouts and sneers, would it not be well if the purveyors of enemy war-cries would ascend to the plane of hard and incontrovertible facts, and state what policy in their opinion the nursing profession ought to pursue? It is extremely doubtful, however, if they are concerned with the well-being of the profession, but rather with the establishing of bubble reputations for themselves. They have no more real desire for the unification of British nurses than Germany has for ensuring to the neutral nations of the world "the freedom of the seas."

IERNE.

